# Postoperative adjuvant therapy for resectable esophageal cancer: A protocol of a systematic review and meta-analysis

**DOI:** 10.1097/MD.0000000000015485

**Published:** 2019-05-17

**Authors:** Tianci Chai, Zhimin Shen, Peipei Zhang, Yuhan Lin, Sui Chen, Zhenyang Zhang, Wenwei Lin, Mingqiang Kang, Jiangbo Lin

**Affiliations:** a Department of Thoracic Surgery, Fujian Medical University Union Hospital; b The Graduate School of Fujian Medical University; c School of Stomatology, Fujian Medical University, Fuzhou, China.

**Keywords:** esophageal cancer, immunotherapy, inhibitors, PD-1 and PD-L1, platinum-based chemotherapy

## Abstract

**Background:**

Esophageal cancer is one of the most common malignant tumors, characterized by early metastasis and high degree of malignancy. Its morbidity ranks 7th among all malignant tumors and its mortality ranks 6th. Postoperative adjuvant therapy after esophagectomy can significantly improve the overall survival rate of patients with locally resectable esophageal cancer. With the breakthrough and progress of immunotherapy, the possibility of cure of esophageal cancer is greatly improved. Some clinical trials have reported that programmed death 1 (PD1) and programmed death ligand 1 (PDL1) inhibitors alone, compared with traditional platinum-based chemotherapy, can benefit patients and effectively extend the overall survival period of patients. We will conduct a systematic review and meta-analysis on the comparison of the efficacy of immunotherapy (PD1 and PDL1 inhibitors) alone and traditional platinum-based chemotherapy, so as to provide a reliable basis for clinicians to formulate the best chemotherapy regimen for patients with esophageal cancer after esophagectomy.

**Methods:**

We will search Pubmed, Medline, Embase, Web of Science, Cancerlit, Google Scholar, and the Cochrane Central Register of Controlled Trials for related studies published before December 1, 2019 without language restrictions. Two review authors will search and assess relevant studies independently. Randomized controlled trials (RCTs) or quasi-RCTs, and prospective cohort studies will be included. We will perform subgroup analysis in sex, age, ethnicity, and tumor stage of esophageal cancer patients.

**Results:**

The results of this study will be published in a peer-reviewed journal.

**Conclusion:**

The results of this systematic review and meta-analysis will provide a basis for clinicians to formulate the best chemotherapy regimen for patients, as well as a research clue for clinical researchers in this field. The results of this study will expand the treatment options for esophageal patients, but due to the nature of the disease and intervention, large sample clinical trials are not abundant, so we will include some high-quality small sample trials, which may cause high heterogeneity.

PROSPERO registration number: CRD42019125000.

## 1 Introduction

Esophageal cancer is one of the most common malignancies with a gradual increase in morbidity, ranking 7th in the incidence and 6th in the mortality of all malignancies worldwide.^[1–3]^ Esophageal cancer is a highly malignant tumor with a strong tendency of invasion and metastasis.^[4–5]^ Despite multiple treatment methods, it is still one of the main causes of cancer-related death in the world.^[6]^ The 5-year survival rate of stage I patients was about 90%, while that of stage II patients was reduced to 45%, that of stage III patients was 20%, and that of stage IV patients was only 10%.^[7]^

Patients with esophageal cancer are usually diagnosed in the middle or advanced stages of tumor. The combination of conventional platinum-based chemotherapy and surgical treatment can significantly improve the overall survival rate of patients, but the prognosis of patients with esophageal cancer is still very poor.^[8–11]^ Immunotherapy is a relatively new field in the treatment of esophageal cancer. Some clinical trials reported that programmed death 1 (PD-1) and programmed death ligand 1 (PD-L1) inhibitors alone have better application prospects than platinum-based chemotherapy.^[12–18]^ We will conduct a systematic review and meta-analysis on the efficacy comparison between immunotherapy and traditional platinum-based chemotherapy, so as to provide a reliable basis for further promotion of immunotherapy and for clinicians to formulate the best chemotherapy regimen for patients with esophageal cancer after esophagectomy.

## 2 Objective

We will assess the efficacy of postoperative platinum-based chemotherapy and PD-1 and PD-L1 inhibitors alone with or without radiotherapy for patients with esophageal cancer.

## 3 Methods

This protocol is conducted according to the preferred reporting items for systematic review and meta-analysis protocols (PRISMA-P) statement.^[19]^ We will report the results of this study adhere to the PRISMA guidelines.^[20]^ This protocol has been registered in the PROSPERO network (registration number: CRD42019125000).

### 3.1 Eligibility criteria

#### 3.1.1 Types of studies

Randomized controlled trials (RCTs) or quasi-RCTs, and high-quality prospective cohort studies published or unpublished will be included, which must have been completed and compared postoperative platinum-base chemotherapy versus PD-1 and PD-L1 inhibitors alone for patients with esophageal cancer.

#### 3.1.2 Types of participants

The participants will be adults diagnosed with locally resectable esophageal cancer histologically or cytologically confirmed who were treated with platinum-based chemotherapy or PD-1 and PD-L1 inhibitors after esophagectomy. No restrictions on sex, ethnicity, economic status, and education will be applied.

#### 3.1.3 Types of interventions

According to the means of postoperative adjuvant therapy for patients with locally resectable esophageal cancer, the trials included will be divided into the following categories.

- Postoperative platinum-base chemotherapy versus PD-1 and PD-L1 inhibitors alone.
- Platinum-based chemotherapy versus platinum-based chemotherapy.

Plus PD-1 and PD-L1 inhibitors

#### 3.1.4 Types of outcome measures

##### 3.1.4.1 Primary outcomes

The primary outcomes will be postoperative overall survival of patients with locally resectable esophageal cancer who were treated with platinum-based chemotherapy or PD-1 and PD-L1 inhibitors after esophagectomy.

##### 3.1.4.2 Secondary outcomes

We will assess the 5-year survival, median survival, recurrence-free survival, complications, and quality of life of patients with locally resectable esophageal cancer who were treated with platinum-based chemotherapy or PD-1 and PD-L1 inhibitors after esophagectomy.

### 3.2 Information sources

We will search Pubmed, Medline, Embase, Web of Science, Cancerlit, Google Scholar, and the Cochrane Central Register of Controlled Trials for related studies published before December 1, 2019 without any language restrictions.

### 3.3 Search strategy

We will use the corresponding keywords or subject terms adhered to medical subject heading terms to search for eligible trials in the databases which were mentioned above without any language restrictions. The Pubmed search strategies are shown in Table 1.

**Table 1:**
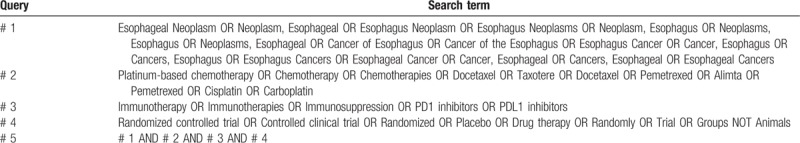
Pubmed search strategies.

### 3.4 Data collection and analysis

We will adopt the measures described in the Cochrane Handbook for Systematic Reviews of Interventions to pool the evidence.^[21]^

#### 3.4.1 Study selection

Two reviewers (CTC, SZM) will investigate each title and abstract of all literatures searched independently and identify whether the trials meet the inclusion criteria as designed and described in this protocol. Two authors (CTC, SZM) will in duplicate and independently screen the full text of all potential eligible studies to exclude irrelevant studies or determine eligibility. The 2 reviewers will list all the studies included and document the primary reasons of exclusion for studies that do not conform to the inclusion criteria. Disagreements between the 2 authors will be resolved by discussing with the third author (JBL), if necessary, consulting with the fourth author (MQK). We will show the selection process in details in the PRISMA flow chart.

#### 3.4.2 Data extraction and management

The 2 authors (CTC, SZM) will extract the following data independently from the studies included.

- Study characteristics and methodology: country, the first author, publication date, study design, randomization, periods of data collection, total duration of study, follow-up duration, and withdrawals, et al.
- Participant characteristics: age, gender, ethnicity, pathology diagnosis, tumor stage, pathologic tumor size, performance status, history of smoking, and inclusion criteria, et al.
- Interventions: therapeutic means, type of operation, extent of resection, drugs, dosage, modality, and frequency of administration, et al.
- Outcome and other data: overall survival, 5-year survival, disease-free survival, median survival, 95% confidence intervals (CIs), recurrence time, quality of life, complications, and adverse events, et al.

We will record all the data extracted in a predesigned table and consult the first author of the trial by e-mail before determining eligibility if the reported data of which are unclear or missing.

### 3.5 Assessment of risk of bias in included studies

Two authors (CTC, ZMS) will use the Cochrane Handbook for Systematic Reviews of Interventions to assess the risk of bias of each study included independently based on the following ranges: random sequence generation (selection bias); allocation concealment (selection bias); blinding of participants and personnel (performance bias); blinding of outcome assessment (detection bias); incomplete outcome data (attrition bias); selective outcome reporting (reporting bias); other bias.^[19]^ Each domain will be assessed as high, low or uncertain risk of bias. The results and details of assessment will be reported on the risk of bias graph. EPOC guidelines will be used to assess the risks of nonrandomized controlled trials included.^[22]^

### 3.6 Data analysis

The data will be synthesized by Review Manager and Stata software. We will conduct a systematic review and meta-analysis only if the data gathered from included trials are judged to be similar enough to ensure a result that is meaningful. The Chi^2^ test and *Ι*^2^ statistic will be used to assess statistical heterogeneity among the trials included in matched pairs comparison for standard meta-analysis. The random effect model will be applied to analyze the data if there is substantial heterogeneity (*P* < .1 or *I*^2^ statistic >50%) and the trials will be regarded to be obvious heterogeneous. Otherwise, we will adopt fixed-effect model to analyze the data. Mantel–Haenszel method will be adopted to pool of the binary data. The results will be reported in the form of relative risk between 95% CI of the date. The continuous data will be pooled by inverse variance analysis method and the results will be shown in the form of standardized mean difference within 95% CI of the date.

#### 3.6.1 Subgroup analysis

If there is high heterogeneity and the data are sufficient, subgroup analysis will be conducted to search potential causes of heterogeneity. Subgroup analysis will be performed in ethnicity, tumor stage, and type of operation.

#### 3.6.2 Sensitivity analysis

Sensitivity analysis will be conducted to assess the reliability and robustness of the aggregation results via eliminating trials with high bias risk.

### 3.7 Publication bias

If there are 10 or more than 10 trials included, we will construct a funnel plot and use Egger test to assess publication bias. If reporting bias is suspected, we will consult the study author to get more information. If publication bias does exist, we will apply the fill and trim method to analyze publication bias in the trials.^[23]^

### 3.8 Evidence evaluation

We will evaluate all the evidence according to the criteria of grading of recommendations, assessment, development, and evaluation (imprecision, study limitations, publication bias, consistency of effect, and indirectness bias). The quality of all evidence will be evaluated as 4 levels (high, moderate, low, and very low).^[24]^

## 4 Discussion

Esophageal cancer is a highly malignant tumor, although there are a variety of advanced treatment methods combined with surgical treatment, but the patient prognosis is very poor. Esophagectomy is still a mainstay in the treatment of esophageal cancer and postoperative adjuvant therapy plays a key role, which is a critical factor contributing to the overall survival of patients. The incidence of esophageal cancer is mainly middle-aged and elderly patients, and the quality of life and physical fitness of postoperative patients are poor. Therefore, therapies that could significantly improve overall survival and have fewer side effects are what we need to pursue now. Immunotherapy is a new field in the treatment of esophageal cancer, and many trials have reported that PD-1 and PD-L1 inhibitors can benefit patients alone more than traditional platinum-based chemotherapy. We will conduct a systematic, comprehensive and objective evaluation of immunotherapy and platinum-based adjuvant chemotherapy. The results of this study will provide a basis for clinicians to formulate the best postoperative adjuvant treatment strategy for patients with esophageal cancer, and also provide scientific clues for researchers in this field.

## Author contributions

Jiangbo Lin and Mingqiang Kang are the guarantors of the article. Tianci Chai and Zhimin Shen conceived and designed the study. Tianci Chai, Peipei Zhang, and Yuhan Lin drafted this protocol. Tianci Chai, Sui Chen, Zhenyang Zhang, and Wenwei Lin performed the search, screening, and extraction. Jiangbo Lin and Mingqiang Kang have strictly reviewed this protocol and approved of publication. Tianci Chai and Zhimin Shen contributed equally to this work.

**Conceptualization:** Tianci Chai, Zhimin Shen.

**Data curation:** Tianci Chai, Zhimin Shen, Sui Chen.

**Formal analysis:** Tianci Chai, Zhimin Shen, Peipei Zhang, Yuhan Lin, Sui Chen.

**Funding acquisition:** Jiangbo Lin.

**Investigation:** Tianci Chai, Zhimin Shen, Peipei Zhang, Yuhan Lin.

**Methodology:** Tianci Chai, Zhimin Shen, Yuhan Lin, Zhenyang Zhang.

**Project administration:** Tianci Chai, Zhenyang Zhang, Jiangbo Lin.

**Resources:** Tianci Chai, Yuhan Lin, Wenwei Lin, Jiangbo Lin.

**Software:** Tianci Chai, Zhimin Shen, Peipei Zhang, Sui Chen, Wenwei Lin.

**Supervision:** Mingqiang Kang, Jiangbo Lin.

**Validation:** Tianci Chai, Zhimin Shen, Peipei Zhang, Jiangbo Lin.

**Visualization:** Tianci Chai, Jiangbo Lin.

**Writing – original draft:** Tianci Chai.

**Writing – review and editing:** Mingqiang Kang, Jiangbo Lin.
